# The Timing of Implant Exchange in the Development of Capsular Contracture After Breast Reconstruction

**Published:** 2008-05-29

**Authors:** Jennifer L. Weintraub, David M. Kahn

**Affiliations:** Division of Plastic and Reconstructive Surgery, Stanford University School of Medicine, Stanford, CA

## Abstract

**Objective:** Capsular contracture is a common complication associated with reconstructive breast surgery. The optimal time interval between the completion of tissue expansion and placement of the permanent implant is arbitrary and incompletely studied in the literature. The aim of the study was to determine whether the time interval between completion of expansion and placement of the permanent implant would affect the incidence of capsular contracture. **Methods:** We conducted a retrospective study of 112 patients with breast cancer, including 140 breasts, who underwent postmastectomy tissue expander placement between 1997 and 2004. All patients underwent replacement of tissue expander with a permanent prosthesis. Data were collected retrospectively, including whether the patient smoked, underwent radiation therapy, had saline or silicone implant reconstruction, required reoperation after tissue expander placement or after permanent implant placement, Baker classification, and the interval between completion of expansion and placement of permanent implant. **Results:** We used a logistic regression model to incorporate the predictors of capsular contracture. Keeping all other predictors constant, we found that the time interval between implant exchange had no effect on capsular contracture. The only significant predictor of capsular contracture was whether the patient required a reoperation after the permanent implant was placed (*P* = .0001). **Conclusions:** Allowing the capsule around a tissue expander to mature does not significantly affect development of capsular contracture. However, a complication that necessitates disrupting the periprosthetic capsule of the permanent implant with an operation significantly increases odds of developing contracture.

In implant-based breast reconstruction, a prosthetic tissue expander is placed in a pocket beneath breast skin and muscle after mastectomy and expanded over a period of weeks to months. Once a goal volume is reached and the soft tissue envelope has been adequately expanded, the temporary expander is removed and a permanent implant is placed. This technique, described independently in 1982 by both Austad and Radovan, uses local tissue to reconstruct the breast, offering the advantage of a color and texture match.^1^,^2^

An inevitable consequence of using breast implants for postmastectomy reconstruction is formation of a periprosthetic capsule. The capsule forms as part of a physiologic response to a foreign body. The periprosthetic capsule is both too large to be digested by the immune system and too inert to elicit rejection.^3^,^4^ Although the capsule initially keeps the implant in its proper position in the breast, over time, many women will experience a hardening and tightening of the capsule known as capsular contracture. Capsular contracture is thought to result from a prolonged process of tissue repair as a response to the presence of a foreign body.^5^ Although surgical technique and the quality of breast prostheses have significantly improved over time, capsular contracture remains one of the most common complications associated with reconstructive breast surgery with an incidence ranging between 0.6% and 30%.^6^–^8^

Although numerous theories have been proposed to explain the causes of capsular contracture, the true etiology has yet to be fully elucidated. Previous studies have shown that certain factors increase the incidence of capsular contracture, including radiation to the prosthesis, infection, contamination with foreign material, location of the implant, and texture of the prosthesis.^9^–^11^ Although the literature is replete with theories regarding the natural history of periprosthetic capsule formation, a factor yet to be adequately evaluated is the timing of implant exchange. The optimal time interval between the completion of tissue expansion and placement of the permanent implant is arbitrary and incompletely studied in the literature.

We theorized that the maturity of the capsule surrounding the tissue expander might affect the formation of capsular contracture associated with the permanent implant. The aim of the study was to determine whether the time interval between completion of expansion and placement of the permanent implant would affect the incidence of capsular contracture.

## PATIENTS AND METHODS

One hundred forty breast reconstructions were studied in 112 breast cancer patients. All patients underwent postmastectomy tissue expander placement followed by replacement with permanent breast prosthesis between 1997 and 2004 at our medical center. All surgical procedures were performed by 1 of 3 reconstructive surgeons. Each of the surgeons utilized a similar operative technique of placing the expander beneath the pectoralis muscle superiorly, with the inferior and lateral aspects of the expander covered by a flap of serratus muscle and/or fascia. Capsulotomy was performed in each patient when the tissue expander was replaced with the permanent prosthesis. A consistency of care was noted among the 3 surgeons with each surgeon uniformly administering antibiotics, applying similar postoperative dressings, and monitoring their patients at similar intervals in the perioperative periods.

Patients were excluded from the study if their follow-up was less than 12 months after placement of permanent prosthesis. Patients were also excluded if tissue expander or implant was permanently removed. Specifically, patients who required removal of expanders or implants because of infection or exposure and *did not* have their prosthetic implant replaced were excluded from the study, as reconstructive course was considered incomplete.

Data were collected retrospectively and maintained in an Excel spreadsheet. Information was collected, regarding potential risk factors in the development of capsular contracture, including whether the patient smoked cigarettes during the reconstructive process or underwent radiation therapy. Radiation therapy as a potential risk factor was further broken down into 3 categories: whether the patient received chest wall radiation more 5 than years prior to tissue expander placement, within 5 years of tissue expansion, or during tissue expansion.

Other information collected included whether saline or silicone implants were used in the reconstruction and the types of complications that required patients to undergo a reoperation after tissue expander placement and/or after permanent implant placement.

Capsular contracture was graded using the Baker classification, measured clinically by inspection, palpation, and by subjective reports of pain from the patient. The Baker level was documented in the clinic notes by the surgeon. Patients with no evidence of capsular firmness or pain were classified as Baker level I. Those with a palpable, firm capsule were classified as Baker level II. Patients with a firm capsule and distortion of the implant were classified as Baker level III. Patients with a firm capsule, distortion of the implant, and with pain were classified as Baker level IV.

Finally, the time interval between completion of expansion and placement of permanent implant was recorded in months.

## RESULTS

The mean age of the patients studied was 48 (range = 25–78 years) years. Our average follow-up was 29 (range = 12–84 months) months after placement of permanent prosthesis. Five percent of patients smoked cigarettes at some point during reconstruction. Sixteen percent (*n* = 23) of patients underwent radiation therapy during reconstruction (Table 1) of the subset of patients who underwent radiation therapy, 17% (*n* = 4) underwent radiation therapy more than 5 years prior to the placement of tissue expander; 17% (*n* = 4) underwent radiation therapy within 5 years after tissue expander was placed; and 65% (*n* = 15) underwent radiation therapy during tissue expansion.

Silicone implants were placed in 46% of patients, whereas saline implants were used in 54%. Ten percent of patients (*n* = 14) developed a complication that necessitated reoperation after placement of tissue expander. These complications included hematoma (*n* = 2); leak/rupture of the tissue expander (*n* = 4); partial necrosis of mastectomy flap with threatened exposure (*n* = 4); seroma (*n* = 1); wound dehiscence with tissue expander exposure (*n* = 2); and infection, including cellulitis (*n* = 1).

**Figure 1:**
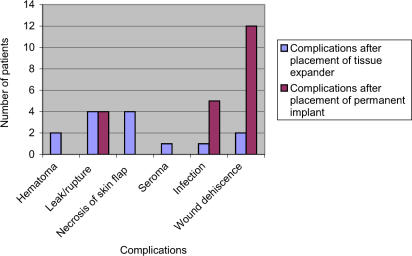
Complications requiring reoperation.

Fourteen percent (*n* = 19) of patients developed a complication (other than capsular contracture) that necessitated reoperation after placement of the permanent implant. These complications included infection/cellulitis (*n* = 5); wound dehiscence with implant exposure (*n* = 12); and implant rupture (*n* = 4) (Figure 1). Eighteen percent (*n* = 25) of patients developed capsular contracture, Baker grades II to IV.

The median time interval for all patients between completion of tissue expansion and placement of the permanent implant was 2.5 (range = 0.5–16 months) months. For the group of patients who *developed* capsular contracture of their permanent prosthesis, the median time interval for implant exchange was 2.5 (range = 0.5–7 months) months. For the patients who did not develop capsular contracture of permanent prosthesis, the median time interval for implant exchange was 3 (range = 0.5–16 months) months.

We used the statistical software program R 2.3.0 to analyze our data. We formulated a logistic regression model that incorporated the potential risk factors to model a binary result, specifically, whether or not capsular contracture grades II to IV occurred. In our model, these potential risk factors were radiation, cigarette smoking, complications that required a reoperation both after the tissue expander was placed and/or after the permanent prostheses were placed, and the exchange interval between completion of expansion and placement of the permanent prosthesis.

Keeping all other predictors constant, we found that the time interval between completion of tissue expansion and placement of the permanent implant had no effect on development of capsular contracture. A shorter exchange interval did not change the odds of developing capsular contracture in our patient population.

In evaluating other potential risk factors for capsular contracture, we found that the *only* significant predictor of capsular contracture was whether the patient suffered a complication significant enough to warrant a reoperation after the permanent implant was placed (*P* = .0001). In our model, keeping all other predictors constant, the odds of developing capsular contracture were 9 times greater if a reoperation occurred after the permanent implant was placed than if a reoperation was not necessary.

Our patients who underwent radiation therapy at any point before or during reconstructive process had the same odds of developing capsular contracture as the patients who did not undergo radiation therapy. We also evaluated radiation exposure as an independent risk factor for the complications that led to reoperation (such as wound dehiscence, infection, and implant rupture). We found that there was no significant difference in the rate of complications in patients who were radiated at any point before or during reconstruction as compared with those who were not radiated.

Similarly, smoking did not significantly affect the odds of developing capsular contracture of the permanent implant.

## DISCUSSION

Reconstructive breast surgery continues to evolve. Advances in techniques of tissue expansion, skin-sparing procedures, and microsurgery have broadened the scope of options for women seeking breast reconstruction. Tissue expansion is a widely used and well-accepted method of breast reconstruction because it utilizes local tissue to safely reconstruct a breast mound. A problem inherent to this method, however, is development of a periprosthetic fibrous capsule that the body produces as a normal response to a foreign body. Hardening of the capsule, or capsular contracture, remains a frequent complication of this modality of breast reconstruction. Contracture can compromise the aesthetic outcome, result in pain, and necessitate further operations.

The etiology of capsular contracture is multifactorial. Whether implants are used in breast augmentation or breast reconstruction adds another level of complexity to the evaluation of what factors influence contracture. Many studies have investigated the relationship between contracture and implant surface texture, bacterial colonization, location of implant placement, and type of implant filler material. There is evidence to suggest that silicone implants with textured surfaces are associated with significantly less capsular contracture than those with smooth surfaces.^6^,^12^–^16^ Although not commercially available for use, long-term studies have shown that polyurethane foam-covered implants may cause the lowest incidence of capsular contracture.^13^,^17^,^18^ Several studies have shown that bacterial colonization of implants is associated with thicker capsules and a higher frequency of capsular contracture.^19^–^21^ Submuscular placement of breast implants has been consistently shown to reduce the incidence of capsular contracture. Placement of implants in the submuscular or subpectoral plane has subsequently become a common practice.^22^–^27^ Studies regarding capsular contracture and filler materials have demonstrated a significantly higher contracture rate using silicone implants than using saline implants when the implants are placed in the submuscular plane.^28^–^31^

What is less clear in the literature regarding factors involved in capsular contracture is the role of capsular maturity. The appropriate interval between completion of tissue expansion and exchange of the permanent prosthesis is essentially unstudied. Some surgeons initially advocated an exchange delay of 6 months after tissue expansion.^32^ This concept was further supported by Holmes' study, in which patients who had a significantly longer exchange interval between expansion and implant placement had lower rates of capsular contracture than patients with shorter exchange intervals.^33^ Subsequently, the preferred time frame for implant exchange was shortened to 2 months on the basis of the notion that the majority of contracture occurred during this time.^32^

Other authors have evaluated capsular maturity by investigating whether the time course of expansion affected capsular contracture. Wickman assessed capsular contracture in women who underwent rapid tissue expansion versus slow tissue expansion, and found no significant long-term difference in breast softness between the groups.^34^,^35^

To our knowledge, the only published study that investigates the timing of implant exchange is by Foo et al, who found that delaying implant exchange has no effect on capsular contracture.^36^ Our study has produced similar results, suggesting that other factors besides the maturity of the tissue expander capsule are more significant in producing capsular contracture of permanent implant.

Our study also showed that complications that occur *after* the permanent implant was placed increased the odds of developing contracture. This echoes other studies that have shown that processes that create inflammation of the capsule predispose to contracture.^37^,^38^ What is unique, however, is that when these same complications occurred while the tissue expander was in place, it did *not* increase the odds of developing contracture of the permanent prosthesis. In other words, inflammatory processes involving the capsule early on do not necessarily predispose to contracture later. However, inflammatory processes later, that is, once the permanent implant is in place, appear to predispose patients to capsular contracture.

This can be explained by 2 hypotheses. First, the nature of the complications after tissue expander placement compared with that after permanent implant placement was not identical in this study population. Complications of seroma and hematoma occurred after tissue expander placement, but not after permanent implant placement. Only 1 patient developed a significant infection warranting a reoperation after tissue expander placement, whereas 5 patients developed significant infection of their permanent implant. This is because the majority of patients who lost their tissue expander because of infection (*n* = 10) opted to either abort the reconstructive process or undergo autologous tissue transfer. These patients were excluded from the study. Therefore, this difference likely reflects a selection bias.

Second, the differences in complications associated with tissue expander placement versus permanent implant placement may reflect the difference in patient-doctor contact in the postoperative period. During tissue expansion, the surgeon sees the patient weekly or bimonthly to proceed with expansion. Complications in this period are likely to be diagnosed early, and serious problems are potentially preempted. In contrast, after the permanent implant is placed, the patient is typically seen less frequently. It is possible that subtle complications, such as subclinical infections, went unnoticed by the patient during the postoperative period, and that complications were more advanced by the time the patient was seen in clinic. More advanced complications may account for the positive correlation with capsular contracture. This enumerates the importance of regular and frequent follow-up visits after the permanent implant is placed.

In contrast to other studies, our patients who underwent radiation therapy at any point before or during their reconstructive process did not have increased odds of developing capsular contracture. This observation has also been noted in other studies. Spears remarked that, although the number of breast reconstruction patients receiving radiation therapy has increased by 15%, he has not noted any increases in the rates of capsular contracture.^39^

One explanation for our finding is the use of improved radiation therapy protocols at our institution that have also been shown to be well tolerated by patients undergoing other modalities of breast reconstruction.^40^ Another explanation for this finding is that in our institution women who have been radiated or anticipate radiation therapy are offered autologous tissue reconstruction. Therefore, only a small percentage (16%) of women in our study underwent radiation therapy. The power of radiation as a risk factor was thus low and may explain why radiation therapy did not increase the odds of developing capsular contracture in our population.

We also evaluated radiation therapy as an independent risk factor for the complications that led to reoperation (such as wound dehiscence, infection, and implant rupture). We found that there was no significant difference in the rate of complications in patients who were radiated at any point before or during reconstruction as compared with those who were not radiated. Again, this is likely explained by both improved protocols for radiation administration and the low number of patients who received radiation, reducing the power of radiation therapy as a predictor of complications.

## CONCLUSIONS

The time interval between completion of tissue expansion and implant exchange does not affect the odds of developing capsular contracture. However, inflammatory complications that necessitate disruption of the periprosthetic capsule of the permanent implant with an operation significantly increase odds of developing contracture. Finally, it is important to note that, although the rate of capsular contracture may not be affected by the implant exchange interval, there may be other reasons to delay implant exchange. The breast soft tissue envelope is expanded to increase its volume, and a recovery period is required to allow soft tissue to recover and reestablish blood supply. Thus, some delay between expansion and implant exchange is necessary to allow this recovery.

## Figures and Tables

**Table 1:** Results

Mean age	48 years
Average follow-up time	29 months*
Patients who smoked cigarettes	5%
Patients who had silicone implants placed	46%
Patients who had saline implants placed	54%
Patients who underwent radiation therapy at some point during reconstruction	16%

*Time period after placement of permanent prosthesis.
